# The blood DNA virome in 8,000 humans

**DOI:** 10.1371/journal.ppat.1006292

**Published:** 2017-03-22

**Authors:** Ahmed Moustafa, Chao Xie, Ewen Kirkness, William Biggs, Emily Wong, Yaron Turpaz, Kenneth Bloom, Eric Delwart, Karen E. Nelson, J. Craig Venter, Amalio Telenti

**Affiliations:** 1 Human Longevity Inc., San Diego, California, United States of America; 2 Human Longevity Singapore Pte. Ltd., Singapore; 3 Blood Systems Research Institute, Department of Laboratory Medicine, University of California San Francisco, San Francisco, California, United States of America; 4 J. Craig Venter Institute, La Jolla, California, United States of America; Plymouth University, UNITED KINGDOM

## Abstract

The characterization of the blood virome is important for the safety of blood-derived transfusion products, and for the identification of emerging pathogens. We explored non-human sequence data from whole-genome sequencing of blood from 8,240 individuals, none of whom were ascertained for any infectious disease. Viral sequences were extracted from the pool of sequence reads that did not map to the human reference genome. Analyses sifted through close to 1 Petabyte of sequence data and performed 0.5 trillion similarity searches. With a lower bound for identification of 2 viral genomes/100,000 cells, we mapped sequences to 94 different viruses, including sequences from 19 human DNA viruses, proviruses and RNA viruses (herpesviruses, anelloviruses, papillomaviruses, three polyomaviruses, adenovirus, HIV, HTLV, hepatitis B, hepatitis C, parvovirus B19, and influenza virus) in 42% of the study participants. Of possible relevance to transfusion medicine, we identified Merkel cell polyomavirus in 49 individuals, papillomavirus in blood of 13 individuals, parvovirus B19 in 6 individuals, and the presence of herpesvirus 8 in 3 individuals. The presence of DNA sequences from two RNA viruses was unexpected: Hepatitis C virus is revealing of an integration event, while the influenza virus sequence resulted from immunization with a DNA vaccine. Age, sex and ancestry contributed significantly to the prevalence of infection. The remaining 75 viruses mostly reflect extensive contamination of commercial reagents and from the environment. These technical problems represent a major challenge for the identification of novel human pathogens. Increasing availability of human whole-genome sequences will contribute substantial amounts of data on the composition of the normal and pathogenic human blood virome. Distinguishing contaminants from real human viruses is challenging.

## Author summary

Novel sequencing technologies offer insight into the virome in human samples. Here, we identify the viral DNA sequences in blood of over 8,000 individuals undergoing whole genome sequencing. This approach serves to identify 94 viruses; however, many are shown to reflect widespread DNA contamination of commercial reagents or of environmental origin. While this represents a significant limitation to reliably identify novel viruses infecting humans, we could confidently detect sequences and quantify abundance of 19 human viruses in 42% of individuals. Ancestry, sex, and age were important determinants of viral prevalence. This large study calls attention on the challenge of interpreting next generation sequencing data for the identification of novel viruses. However, it serves to categorize the abundance of human DNA viruses using an unbiased technique.

## Introduction

Research on the human microbiome has been primarily directed to the prokaryotic composition of the human microflora. Because most of the analyses use 16S rRNA gene-based amplification, the viral content has been rarely captured in large-scale microbiome studies. In contrast, analysis of the whole human genome by next-generation sequencing is an exercise in metagenomics: after mapping sequencing reads to the human reference genome, there is a significant proportion (generally 5% of all sequence data) that is left uncharacterized [1]. Bacterial but also archaea, non-human eukaryotic and viral sequences are thus a by-product of the sequencing of the human genome.

Previous studies of the human virome have addressed the viral component of the gut flora [2–4] and skin [5–7], with particular attention to the very abundant bacteriophages [7, 8]. A thorough review has been published recently [9]. Many viruses are present in peripheral blood—in particular, members of the *Herpesviridae* and *Anelloviridae* families are identified in the absence of disease. Metagenomic studies on blood have identified great genetic diversity of anelloviruses [10–12]. Metagenomic studies also lead to the identification of novel RNA viruses—for example the identification of two rhabdoviruses [13]. Other viral sequences in the blood of healthy individuals are related to members of the Picornaviridae, Poxviridae, Flaviviridae, and Phycodnaviridae families (reviewed in [9]). Finally, a number of viruses, prominently retroviruses, are integrated in the human genome as provirus, while others may integrate occasionally or accidentally [14].

The study of the human virome is particularly relevant in the context of current discussions of next-generation sequencing for surveillance of viruses in blood and for transfusion safety [11, 15, 16]. Only viruses that are both pathogenic and transfusion-transmissible are routinely tested for and excluded from blood-derived products. Rejecting all virus-infected donations irrespective of pathogenicity would not be sustainable as most donors are anellovirus positive. The time required to develop and implement specific virus nucleic acid tests to emerging viral pathogens in the blood supply has greatly improved as seen with the response to recent Zika virus outbreak [17]. Exclusionary steps for viruses can also vary depending on the recipients in whom sequelae may vary in severity such as the use of parvovirus B19-reduced plasma pool to derive products for pregnant B19 seronegative women and immunocompromised patients. Seasonal variation in virus prevalence can also affect when testing is implemented such during mosquitos season for West Nile virus RNA. As the rate of human genome and associated DNA viruses sequenced from blood continues to grow data a baseline will be available to compare rates of infections with various DNA viruses, as described in this study, to that in future populations.

There are many open questions on what could be considered a “normal” human blood virome. Recently, the National Heart, Lung, and Blood Institute of the National Institutes of Health convened a working group on the microbiome that identified studies of the human virome a key priority [18]. The present study aims at establishing the DNA virome in over 8,000 individuals participating in a large-scale sequencing effort of the whole human genome [1]. A careful definition is key to diagnosing infections, to understanding the role of the virome in chronic disease, and for settling claims for the identification of new viral species in humans.

## Results

### Viral sequences in the unmapped reads

We sequenced the genomes of 8,240 individuals. On average, each sequencing reaction generated 1 billion reads. The total input approached 1 PB. The majority (95%) of reads were successfully mapped (S1 Fig) to the human reference genome GRCh38 (hg38). Among the remaining reads, similarity search assigned 9% to non-reference human sequences, 1% to other primate sequences, 0.2% to bacteria, and 0.01% to viruses. The bulk of unmapped reads mainly represents reads with multiple mappings to the human reference, but also microbial genomes absent in the database, and low quality reads.

**Fig 1:**
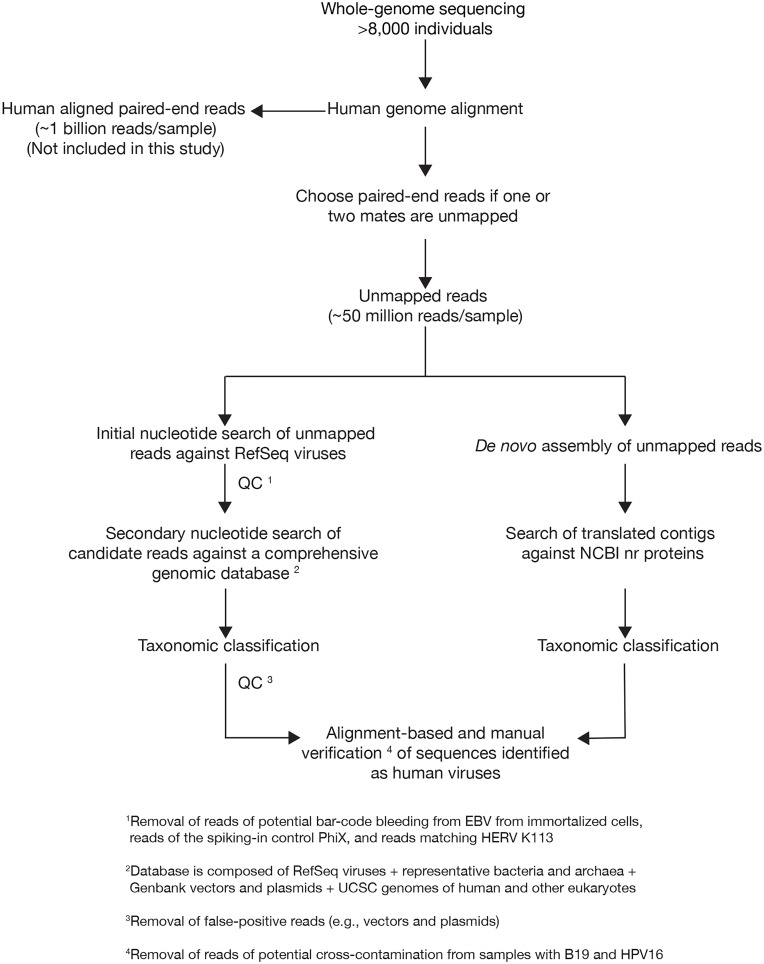
Study design. The flowchart summarizes the steps followed to identify viral content in the human blood DNA from whole-genome sequencing reads.

**Fig 2:**
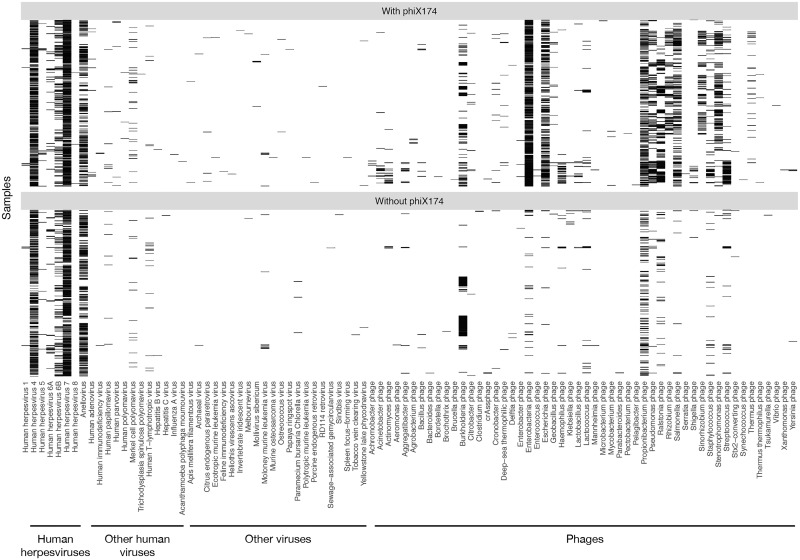
Viral content. The heatmap shows the presence of reads of viral nature in sequencing reactions of blood from 8,240 individuals. Extensive phage and other viral DNA is found in sequencing reactions, but it is almost universally associated to including phiX174 phage spike-in in the reaction (used in 60% of samples). For reference, we include the ubiquitous identification of human endogenous retrovirus (HERVs) sequences in the pool of unmapped reads.

We launched 0.5 trillion similarity searches against the NCBI viral genomes (Fig 1). This step mapped sequences to 94 viruses (S1 Table). Samples carried a median of about 400,000 viral reads. However, the majority corresponded to phiX174, used as spike-in control in the sequencing process, or to human endogenous retroviruses (HERV) that are discarded during alignment (Fig 2). Samples that carried phiX174 were also enriched in reads from multiple phages, which we interpret as contamination of the commercial preparation of phiX174. Epstein-Barr virus (EBV, HHV4) reads were abundant in sequences of the human reference genome NA12878 (www.nist.gov/programs-projects/genome-bottle) and in a subset (n = 148) of participant samples where the input DNA material was, in retrospect, from cell lines that use EBV in the process of cell immortalization. Furthermore, we observed cross-contamination from the EBV content in the human genome immortalized cell line NA12878 to other samples on the same flow cell (S2 Fig). The human reference genome NA12878 is used as standard reagent in sequencing laboratories.

In a second step, viral candidate reads were searched against a comprehensive database of viruses, vectors, bacteria, archaea, human, and other eukaryotes to reduce false-positive matches from the initial search. We identified 11% reads that would result from plasmid sequences engineered with sequences such as viral promoters. Therefore, we removed from downstream analysis reads of phiX174 and associated contaminant phages, HERVs, reads from samples containing EBV used in cell immortalization, and EBV reads from samples that were potentially contaminated and plasmids and vectors. Flow cells with high-titer samples of human papilloma virus (HPV) and parvovirus B12 contained other positive samples that were potential false positives (S3 Fig). Single indexing, where the barcodes are embedded in one of the sequencing library adapters, comes with a risk of misidentification of sequences sharing flow cells [19]. The quality control steps are depicted in Fig 1.

We compared the sensitivity of detection of viruses using nucleotide-based search with individual reads versus using protein-based search after *de novo* assembly of reads into contigs and translation (Fig 1 and S4 Fig). The mapping of single reads identified 19 human viruses. In contrast, contigs could only be assembled for 8 viruses because it required the presence of 1 to 4 orders of magnitude more viral reads in the sample (S4 Fig). Overall, viruses were detected by both read mapping or contigs in 137 samples, and only by read mapping in 3,342 samples. Because of the low sensitivity of the approach using contigs, the study proceeded using individual reads.

While it would have been ideal to perform a complete search of translated read-to-translated NCBI nt database using tools such as TBLASTX, this approach would be prohibiting in terms of computational demands. of translated read-to-translated NCBI nt database using tools such as TBLASTX, this approach would be prohibiting in terms of computational demands.

### Human DNA virome

**Fig 3:**
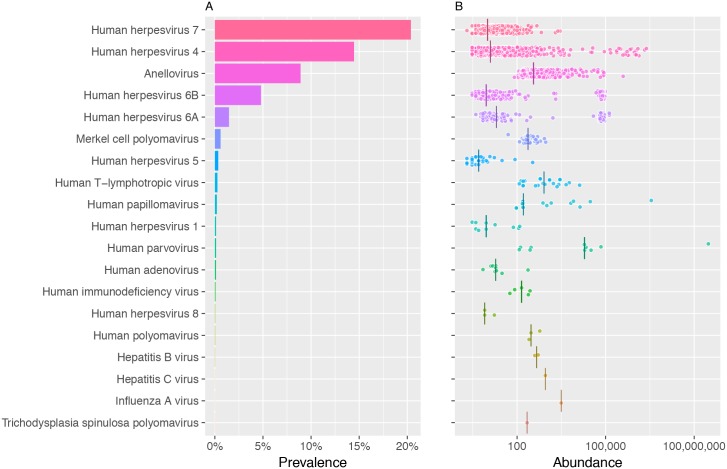
Prevalence and abundance of human DNA viruses and retroviruses in 8,240 individuals. **A.** Frequency of 19 human viruses in the study population ranked according to their prevalence. **B.** The viral load of human viruses represented on the x-axis as genome copies per 100,000 human cells; the bar represents the median.

Among the 94 different viruses identified in the study materials, we identified viral reads for 19 human viruses (Fig 3 and Table 1). Among the herpesvirus (HHV), HHV7 was found in 20%, and EBV was identified in 14% of the individuals. Analysis of sequence diversity identified the presence of both EBV subtypes 1 and 2. The estimated proportion was 80% for subtype 1 and 20% for subtype 2, consistent with previous knowledge [20]. HHV6A and HHV6B were identified in 1.5% and 5% of individuals, respectively. We identified fewer individuals carrying sequences of other human herpesviruses: Herpes simplex 1 (HSV1), Cytomegalovirus (CMV, HHV5), and HHV8.

**Table 1:** Detected human viruses in blood DNA of 8,240 individuals.

Virus	Number^1^ of individuals	Percentage of individuals	Number of sequencing reads per individual		Abundance^2^ of viral genomes per individual		Coverage of viral genome	
			Median	Maximum	Median	Maximum	Minimum	Maximum
Human herpesvirus 7 (HHV-7)	1,678	20.37%	2	702	10	2,860	0.001	0.688
Human herpesvirus 4 (HHV-4, EBV)	1,190	14.45%	4	732,061	12	2,404,531	0.001	637.338
Anellovirus (TTV & TLMV)	734	8.91%	2	2,416	359	392,179	0.046	110.236
Human herpesvirus 6B (HHV-6B)	395	4.80%	2	26,738	9	97,274	0.001	24.74
Human herpesvirus 6A (HHV-6A)	121	1.47%	6	38,254	20	134,595	0.001	36.016
Merkel cell polyomavirus (MCPvV)	49	0.59%	2	8	236	935	0.028	0.223
Human herpesvirus 5 (HHV-5, CMV)	29	0.35%	2	106	5	338	0.001	0.067
Human T-lymphotropic virus (HTLV-1/2)	22	0.27%	13	131	820	13,143	0.034	2.251
Human papillomavirus (HPV)	17^3^	0.19%	2	106,590	162	3,521,083	0.02	2,179.46
Human herpesvirus 1 (HHV-1, HSV-1)	10	0.12%	2	34	9	123	0.001	0.034
Human parvovirus B19	10^4^	0.12%	167	2,841,285	19,298	302,149,810	0.028	78,459.64
Human adenovirus	9	0.11%	1	11	19	235	0.004	0.046
Human immunodeficiency virus (HIV-1/2)	5	0.06%	2	3	142	275	0.015	0.046
Human herpesvirus 8 (HHV-8, KSHV)	3	0.04%	2	4	8	17	0.002	0.004
Human polyomavirus	3	0.04%	2	4	297	588	0.061	0.122
Hepatitis B virus (HBV)	2	0.02%	3	4	460	521	0.093	0.186
Trichodysplasia spinulosa polyomavirus	1	0.01%	2	2	219	219	0.057	0.057
Hepatitis C virus (HCV)	1	0.01%	18	18	912	912	0.286	0.286
Influenza A virus	1	0.01%	4	4	3,212	3,128	0.584	0.582

^1^ Some individuals may carry more than one virus.

^2^ Abundance is estimated per 100,000 human cells.

^3^Four samples possibly due to cross-contamination.

^4^ Four samples possibly due to cross-contamination.

We identified a significant presence of anelloviruses (Torque teno virus [TTV] and TTV-like mini virus [TLMV]) in 9% of the individuals. Other viruses were identified in less that 1% of the study population (Fig 3 and Table 1). We took interest in the presence of sequence reads for papillomavirus (7 different types: 2, 10, 16, 92, 137, 163, and 179) in 17 individuals. Upon validation, we identified a cluster of individuals with the oncogenic type 16 in the same flow cell. We identified the wrong inclusion of a tumor sample in the analysis. This sample corresponded to a head and neck tumor containing large presence of papillomavirus 16 that led to contamination of samples sharing the same flow cell. Parvovirus B19 was identified in 10 individuals; however, four positive samples shared the flow cell with the sample with the highest load of viral copies (> 300 million viral copies/100,000 cells) and where thus classified as contaminants.

**Fig 4:**
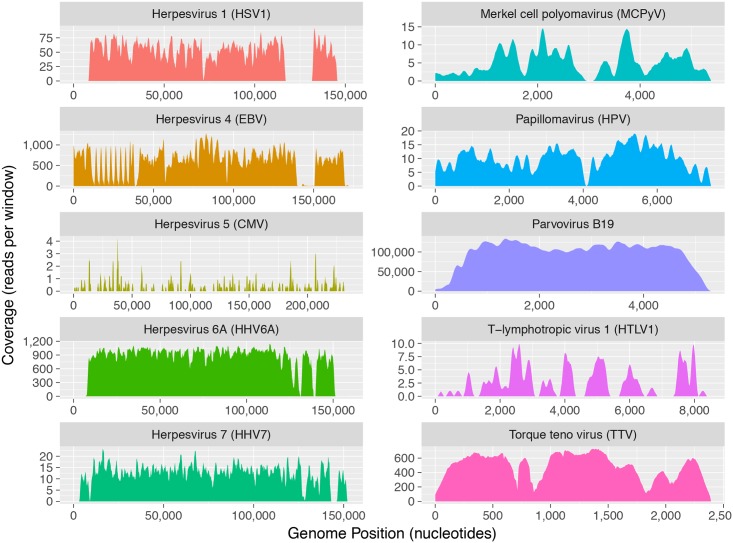
Genome coverage of selected human viruses. Shown are the alignment of reads contributed by all individuals carrying the corresponding virus. The depth of coverage (y-axis) changes in scale as a reflection of the viral abundance and prevalence. Gaps in coverage (e.g., in EBV) generally reflect repetitive regions that are masked during data processing.

We aimed at reconstructing viruses across many samples (Fig 4). The purpose of this step is to provide proof that the viral presence is confirmed by demonstrating broad and average coverage of each viral genome, and not the result of skewed accumulation of local reads—for example at CMV promoters in plasmids. It also offers a detailed view on viral polymorphism and subtypes. This was done for viruses with enough reads or present in numerous individuals, where we could reconstruct the viral genomes with significant coverage (Fig 4). For viruses where only a few reads could be identified, we checked them manually for unambiguous mapping.

### Viral integration

**Fig 5:**
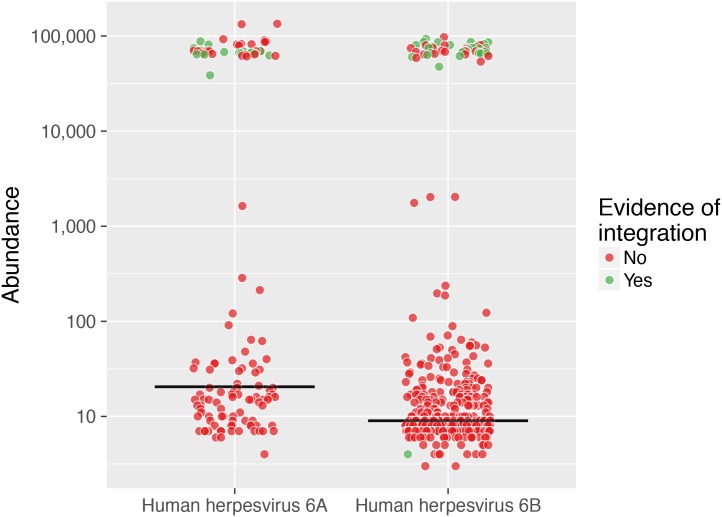
Integration of human herpesvirus 6. The two populations of HHV6A andHHV6B are present in a bimodal distribution. The frequency of integrated viruses, at approximately 0.5 per cell corresponds to the haploid nature of the integration in the case of inherited, vertical transmission—from one of the parents. The identification of chimeric reads, or paired human-virus reads is shown for a substantial proportion of integrated HHV6 (green dots). The bar represents the median.

HHV6 can integrate in the human genome in telomeric regions and can be inherited through the germline [21]. We identified integrated HHV6A/B in 0.5% of the individuals. Fig 5 depicts the expected binomial distribution where samples with integrated copies have 100, 000 viral copies per 100,000 human cells (one integration event in every cell). The precision of this number attests to the highly quantitative nature of the sequencing protocol. Actual proof of integration was also obtained for most of those samples though the identification of chimeric reads or virus-host paired reads (Fig 5). In contrast, samples without integration have 3 to 4 orders of magnitude lower abundance.

Other than the integration events of HHV6 –and the presumed events (insufficient reads to identify the integration site) for human immunodeficiency virus (HIV) and human T lymphotropic virus (HTLV1/2)–we did not have direct proof for other integrated viruses. However, we identified two individuals carrying DNA sequence reads of RNA viruses, influenza and hepatitis C virus (HCV). In the first individual, we observed 4 reads of influenza virus. The reads were mapped to different regions of the viral matrix genes (M1 and M2) (S5 Fig), as well as in the terminal read, a short plasmid tail representing the cloning site of common vector backbones. A possible explanation is that this individual received a DNA-based vaccine. In the second individual, we identified 18 HCV reads. The resulting sequence is similar to HCV clone from Pakistan, which coincides with the demographic information on the presumed carrier (S5 Fig).

An additional sample contained many paired-end chimeras between CMV and human chromosome 11 and 15. Closer inspection revealed a lack of coverage of the CMV genome, with a large number of reads uniquely mapping to CMV regulatory elements used in expression vectors [22]. A similar situation was found in a sample that contained many reads of SV40 of plasmid origin.

### Giant viruses and other viruses of interest

We identified a few viral sequences of Mollivirus in 8 individuals with a median of 2 reads per sample, Paramecium bursaria Chlorella virus in 3 individuals with a median of 2 sequence reads per sample, Apis mellifera filamentous virus in 2 individuals with a median of 2 sequence reads per sample, Melbournevirus in 2 individuals with a median of 3 sequence reads per sample, and Acanthamoeba polyphaga moumouvirus in 1 individual with 2 sequence reads.

We observed the presence of occasional reads with correct match to animal retroviruses (Fig 1): Feline immunodeficiency virus and RD114 feline retrovirus, Ecotropic, Polytropic and Moloney murine leukemia virus, and Porcine endogenous retrovirus. The source of these viruses is likely to be through contamination of cell lines or the environment [23, 24].

We identified in a single individual the presence of 8 reads (abundance = 2,432 particles) of a virus corresponding to the sewage-associated gemycircularvirus. This virus was also identified in transfusion plasma pools and clinical samples [16], thus raising awareness for the possibility of gemycircularviruses infect humans or alternatively, reflecting contamination occurring during phlebotomy or plasma pool processing.

We identified a few viral sequences of archaeal viruses (Archaeal BJ1 virus and Halovirus) in 4 individuals with a median of 9 reads per sample. There is debate in the literature whether these viruses should be referred to as phages [25], and there is no sufficient information on whether archaea, and thus their viruses, may represent actual flora of humans [26].

### Associations with sex, ancestry and age

**Fig 6:**
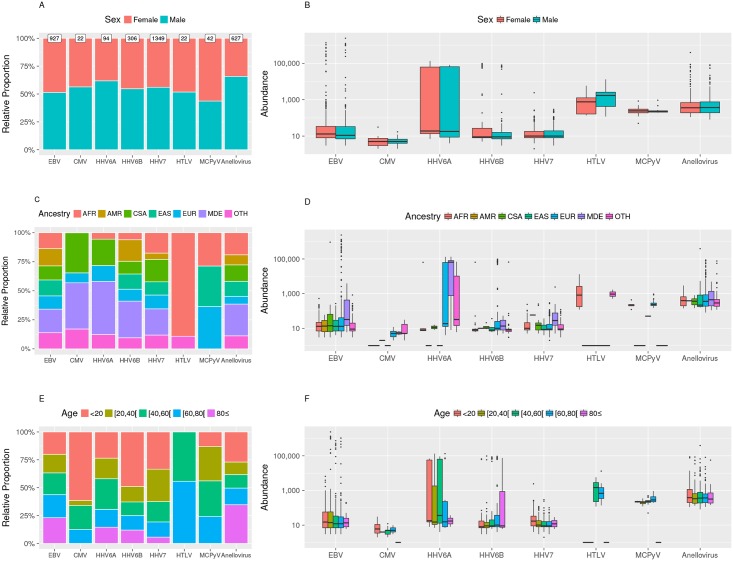
Relative proportion and viral load in the context of age, sex and ancestry. The relative proportion, normalized to 100% for visualization purposes (A, C and E) and distribution of observed viral loads (B, D and F) are depicted for the 8 viruses that have the largest prevalence in the study. Among the 4,505 with demographic information, the ancestries were: EUR, European = 3,048; AFR, African = 665; MDE, Middle Eastern = 94; EAS, East Asian = 91; CSA, Central South Asian = 54; AMR, Admixed American = 8; Multi-Racial and Others = 545.

Complete demographic information was available for 4,505 individuals. We observed a greater prevalence of circulating viruses in men than in women (Fig 6 and S6 Fig). We also observed difference in viral prevalence in relation to age and ancestry (S6 Fig). Deltaretroviruses were predominantly identified in individuals of African ancestry from different geographical locations. Twenty out of 22 human T-lymphotropic virus (HTLV) infections (90%) were HTLV-2. CMV, HHV6A and B and HHV7 were more prevalent in the younger groups, with higher loads of HHV7 identified in them (Fig 6). Statistical significant differences for demographic characteristics and viral prevalence or viral load are summarized in S2 Table. Overall, viral presence associated with age (p-value = 5.6e-25) after adjustment for ancestry (p-value = 1.3e-20) and sex (p-value = 1.4e-9); (S6 Fig).

## Discussion

The current work defines the human DNA blood virome in more than 8,000 individuals that we consider as representing a general population. The study leverages sequencing of the human genome that generates approximately 5% of reads (the sequence of a fragment of the genome) that do not map to the human reference genome. This large pool of reads primarily includes unmapped and repetitive human reads, bacterial reads, but also lesser numbers of sequences from archaea, eukaryotes, and viruses [1]. We identified 94 different viruses, including human DNA viruses, however, the pools of non-human reads are known to contain contaminant DNA from reagents [27, 28]. The routine process of sequencing human DNA does not capture RNA viruses except through the identification of proviruses and other possible viral integration events.

Among sequences that mapped to 94 viruses, we identified 19 human viruses in 42% of the study participants. In addition to a wide representation of human herpesviruses and anelloviruses, the study identified 7 different papillomavirus types, including the oncogenic type 16, HIV, HBV, 3 different polyomavirus types and parvovirus B19. These viruses generally correspond to those known to be highly seroprevalent in the human population [29]. Viral sequences in the study represent a concentration of two to millions of genome copies per 100,000 cells.

We identified sequences of most members of the herpesvirus with the notable exception of Varicella-Zoster virus. This virus is easily identified in blood from immunosuppressed hosts and in immunocompetent subjects with active herpes zoster disease [30, 31]. It is however reported absent in blood in the immunocompetent host [32]. We also observed papillomavirus reads in 0.2% of the study participants. Papillomavirus DNA was previously identified via PCR amplification in 8.3% (15/180) of healthy male blood donors [33]. The Merkel cell polyomavirus (MCPyV), found in 0.55% of the study participants, is highly seroprevalent in the population [34]. MCPyV was reported in 22% of blood samples from healthy donors using PCR [35]. We also identified Trichodysplasia spinulosa polyomavirus (TSPyV) [36], which is also seroprevalent in humans [37]. TSPyV viremia has been described, via PCR amplification, in immunosuppressed individuals but not in healthy controls [38].

The presence of viruses in blood products can be relevant for transfusion medicine. Currently, laboratory testing of donated blood prior to transfusion includes screening of HIV-1 and HIV-2, HTLV-1 and 2, HCV, HBV, West Nile virus, and Zika virus. The clinical impact, if any, of transmission of the highly prevalent GBV-C (aka pegivirus A) and of anelloviruses, is to be deciphered [39, 40]. Parvovirus B19 [41] and other parvoviruses [42] are of concern to transfusion safety because these viruses are not routinely screened for and they lack a lipid envelope, rendering pathogen inactivation procedures less effective. The observation of other human DNA viruses in the study population—for example HPV, MCPyV, HHV8 and adenovirus—adds to the list of viruses that could be potentially transmitted via blood products [43].

The coverage (30X) required for sequencing of the human genome [1] limits the ability to map integration events. This would rely on abundance of sequencing paired reads that encompass viral and human sequences. However, integration into the human genome was observed for HHV6A and B, known to occur in about 0.5% to 1% of humans [44, 45]. Integration by RNA viruses (other than retroviruses) has been described occasionally [14], and we were intrigued to identify one individual carrying few sequence reads of influenza virus that we attributed to the possible use of a DNA-based influenza vaccine (because of the presence of a small plasmid fragment in the sequence). The second surprising event was the identification of multiple sequence reads of HCV matching to viral clones from Pakistan, in an individual from the same geographical origin. There has been discussion on the role of reverse transcriptase activity determining the accidental integration of viral RNA in the genome [46], and specific to HCV, the occasional claim of integration [47].

Younger study participants were more likely to have human viruses identified in blood—which is consistent with the impact of seroconversion window at younger age. Differences in viral prevalence and type of virus varied also by ancestry: geography and local epidemiology may be the driving epidemiological factor. We observed an unexpected bias towards greater prevalence of circulating viruses in men than in women that remained significant after adjusting for the other demographic factors. There have been many descriptions on differences in prevalence, susceptibility to infection and disease severity across sex. The current thinking is that females tend to mount higher innate, cell-mediated, and humoral immune responses than males [48].

Next-generation sequencing is used for the discovery of new human pathogens—particularly in the setting of acute infection. Although we identified 94 different viruses, we found that large numbers of viral sequences represented contamination. Specifically, we observed a very significant presence of phage DNA associated with use of phage phiX174 used to allow real-time quality metrics during sequencing. Although there is a possibility that some phage DNA could translocate from the gut [49], the presence of other phages and viruses each time that phiX174 was used is revealing of intrinsic contamination of the commercial phiX174 materials. Phage DNA can also derive from bacteria contaminating the reagents [27, 50]. Beyond phages, there are reports of false-positive results and claims of viral pathogen discovery traced back to specific steps in the process of sequencing; for example, the identification of parvovirus-like sequences in nucleic acid extraction columns [51, 52] or Moloney MuLV genome in cancer cell lines [53]. Therefore, the presence of a novel DNA virus in blood would require the use of numerous control experiments to exclude contamination. More generally, we identified animal retroviral sequences that likely reflect the contamination of cellular reagents or from environmental sources—a critical consideration given the past history of claims such as with Xenotropic murine leukemia virus-related retrovirus (XMRV) that was reported to be associated with prostate cancer and chronic fatigue syndrome. A massive effort was required to reverse those claims [54]. Finally, many reads were falsely attributed to viruses due to contamination with plasmid sequences that use viral regulatory cassettes.

We evaluated the presence of the recently discovered giant viruses [55]. Our finding of a small number of reads in only 0.2% of the study population suggests that giant virus DNA is not a frequent finding in blood or that its detection also reflects reagent or laboratory contamination [56]. In addition, the presence of samples with high viral-titers leads to misidentification of samples, due to sharing of barcodes in single-index sequencing libraries [19]. This problem has also been described as “sample bleeding” that refers to the incorrect assignment of reads to multiplexed samples that are being sequenced in the same sequencing lane [57]. Dual-indexing will be needed for more accurate studies of the human virome. Many of the observed viruses might be truly present in human blood—however, it is difficult to distinguish them from prevalent contaminant viral sequences. Study design, epidemiological setting and downstream validation by independent techniques are needed to propose novel viruses. Overall, the analysis aims at defining the normal DNA virome background in blood in a presumably healthy population against which novel discoveries can be proposed.

This study has the following limits. It analyzes a convenience population that does not contribute specific data on infectious diseases. However, this can be seen as an advantage in terms of better representing a general population. The nature of the sequencing protocol implies limited amplification of the viral genetic material, and a significant competition from the larger human genome. Therefore, this approach may not identify lower concentration viruses that could be revealed by using viral particles enrichment [58, 59] or viral genome capture [60, 61]. The latter methods rest on the ability to capture closely related sequences by hybridization to short conserved probes. Other recent approaches include methods that enable human viral epitope-wide exploration of immune responses in large numbers of individuals. This latter approach is effective for determining past viral exposure [62]. The study was not conceived for the discovery of highly divergent, novel human viruses, as this requires the use of less stringent similarity criteria for detecting divergent (relative to those already known) viral sequences. Lastly, the study did not address the RNA virome in human blood. Thus, the highly prevalent blood-borne RNA pegivirus A (GBV-C) in the *Flaviviridae* family was not detected here.

The interest of the study derives from the size of the investigation that serves to define the human DNA blood virome. The second, and equally important part of the study is the description of the contamination profile during genome sequencing that may confound the discovery of novel human viruses. Increasing numbers of humans undergoing whole genome and transcriptome sequencing will support the precise description of the human blood DNA and RNA virome.

## Materials and methods

### Study characteristics

Participants were representative of the spectrum of age (between 2 months and 102 years with a median of 56), and of major human populations and ancestries. Specifically, the study included EUR, European = 5,384; AFR, African = 1,049; MDE, Middle Eastern = 213; EAS, East Asian = 159; AMR, CSA, Central South Asian = 94; Admixed American = 16; and Multi-Racial and Others = 1,325. The study population was not ascertained for a specific infectious disease status. Other aspects of the study and the performance of genome sequence are detailed in Telenti et al. [1].

### Ethics statement

New (Western Institutional Review Board, www.wirb.com) and existing IRB-approved consent forms for participation in research and collection of biological specimens and other data used in this publication were reviewed and confirmed to be appropriate for use. All adult subjects provided informed consent, and a parent or guardian of any child participant provided written informed consent on their behalf.

### Sequencing

Library preparation was carried out using the TruSeq Nano DNA HT kit (Illumina Inc.). Libraries were combined into 6-sample pools and clustered. Flow cells were sequenced on the Illumina HiSeqX sequencer utilizing a 150 base paired-end single index read format. Despite of the use of TruSeq technology, several ssDNA viruses were identified. It is possible that this is a reflect of extensive secondary structure of the naked viral DNA [63] and of replicative intermediate forms that are dsDNA [64].

### Identification of unmapped sequences

For each BAM file, we extracted read pairs with either one or both of the reads not mapping to hg38 using sambama [65] with filtering for “unmapped” or “mate_is_unmapped”. Read pairs with average base quality below 30 were removed. Read pairs with low complexity identified using String Graph Assembler [66] with the following parameters dust-threshold = 2.5 and quality-filter = 50 then they were removed. Samples with more than 10% unmapped reads were excluded from further analysis.

### Identification of viral sequences

Unmapped reads were in a first step searched for putative viral matches by blastn [67] against the NCBI RefSeq [68] viral reference genomes (> 8,000 viruses and phages) [69] using an *e*-value ≤ 1e-10. In a second step, candidate reads with viral hits were searched against a more comprehensive database comprised of NCBI RefSeq genomes of viruses, representative bacteria (1,636 species and strains), archaea (389 species and strains), and fungi (two species), and UCSC genomes of human, chimp, mouse, chicken, and fruit fly, and NCBI nt vectors (274,565 sequences) and plasmids (778 sequences) using blastn with *e*-value ≤ 1e-20. Viral hits were filtered for bit-score ≥ 190. Reads with hits other than viruses with bit scores greater than or equal to the viral hits were discarded. Finally, randomly selected reads with viral hits of the human viruses were manually and visually verified by searching (blastn) against NCBI nt (online) and by aligning the reads to the corresponding viral genomes.

### Estimating viral abundances

The normalized abundance of a virus in a sample was estimated in genome copies per human cell (viral genomes per human diploid genome) with the following equation:

$$virus\;abundance=\frac{2\times \frac{number\;of\;reads\;mapped\;to\;viral\;genome}{virus\;genome\;size}}{\frac{number\;of\;reads\;mapped\;to\;human\;genome}{human\;genome\;size}}$$

For ease of interpretation, values are referred to a “viral copies per 100,000 human cells”. The fraction of viral reads has been shown to generally correspond to its viral load as determined by real time PCR [3, 58, 70].

### Assembly of unmapped reads

The unmapped reads were also assembled in contigs using SOAPdenovo [71, 72] with *k*-mer size 91 for each sample. Contigs that were mapped to the human reference with > 90% identity on > 30% length were removed. The remaining contigs were then mapped to the hg38 regions that were masked as repeat in UCSC goldenPath using blastn [67] without low complexity filtering to remove contigs that contain > 20% repeat sequences. Contigs passing the above filtering steps were clustered into non-redundant set using CD-Hit [73, 74] with 90% global identity threshold. Non-redundant clusters were searched for matches to viral proteins using DIAMOND [75] against NBCI non-redundant proteins (nr).

### Prediction of integration sites

To detect potential cases of integration between the viral genome and the human genome, identified viral reads were aligned to a database comprised of the viral genomes and the human reference genome hg38 to detect potential cases of integration, which were predicted via the identification of chimeric reads and chimeric mates using BWA [76] with the maximal exact matches algorithm “bwa mem”. An integration event was predicted when either one mate of a paired-end read aligned to a virus genome and the other mate aligned to the human genome or a single mate chimerically split into two alignments where one part mapped to a virus genome and the other part mapped to the human genome.

### Association with demographic characteristics

We conducted a logistic regression analysis under a generalized linear model (GLM) with binomial distribution for the presence of human viruses in response to the individuals’ sex, ancestry, and age along with the cohort information as the covariate using the ‘glm’ method in R, followed by the `step`method for identifying the optimal model. The significance of the interactions was determined by chi-squared tests for the deviance table of the GLM. Statistical significances of the differences in prevalence and abundance across the demographic characteristics for each virus were estimated using chi-square test and Kruskal-Wallis test, respectively, followed by multiple test correction for the generated *p*-values.

### Data access

Virome reads are available for downloading at www.HLI-OpenData.com/Virome/. In addition, see the Data Access Statement (www.humanlongevity.com/wp-content/uploads/HLIDataAccessAgreement020416.docx.) for information on extended access.

## Supporting information

S1 Fig Read mapping statistics. Unmapped reads in deep sequencing of the human genome using Illumina HiseqX10 technology. The average percentage of unmapped reads per sample is around 5.23%, and median is 4.91%.
(TIF)

S2 Fig Abundance of EBV in association with use of human reference genome NA12878. The distribution of the abundance of EBV is shown for the EBV B95-8 strain-immortalized the cell line of NA12878, for samples sequenced sharing the same flow cell with human genome NA12878 and for samples sequenced in the absence of human genome NA12878 in the sequencing flow cell. We used the conservative approach of eliminating all the positive samples from flow cells containing NA12879 because the high counts indicated that most samples were contaminated. Only a minority of samples had low counts, and we did not attempt alignment to the EBV B95-8 genome because of the few available reads. The bars represent the median.
(TIF)

S3 Fig Distribution of samples with viruses across the sequencing flow cells. The number of viral reads per samples are shown on the y-axis in relation to the number of samples per flow cell that are positive for the corresponding virus. The presence of multiple positive samples in flow cells that contain one high viral-titer sample is suggestive of contamination by misidentification by sharing of barcodes in single-index sequencing libraries. The bars represent the median.
(TIF)

S4 Fig Assembly of contigs of human viruses. The sensitivity of identification of human viruses differs when using contigs from de novo assembly of reads, versus using individual reads. The upper panel is based on raw counts of the virus reads and the lower panels show the normalized viral abundances. The identification of viruses is improved by several orders when using read mapping. However, excessive number of reads (depth) may lead to failure of the assembly process Overall, viruses were detected by both read mapping or contigs in 137 samples, and only by read mapping in 3,342 samples. It came as a surprise that in 13 samples the identification of viral sequences (anellovirus, CMV, and HIV) was achieved using only contigs. After manual inspection, the CMV and HIV contigs represented plasmids sequences. Eleven samples with anelloviruses, represented by four clusters, were detected by contigs only because the individual reads had low identity (less than 70%) with the corresponding virus reference genome indicating the presence of divergent anelloviruses. Specifically, two contigs had the closest match as TTV-like mini LY1, one contig had the closest match as Torque teno mini virus 3, and one contig had the closest match as unclassified Anelloviridae isolate TPK01. The bars represent the median.
(TIF)

S5 Fig Sequence reads from RNA viruses. Panel A depicts the alignment of 4 reads from one individual to the influenza H1N1 reference sequence M1 and M2, segment seven. Closest match; serotype = H1N1, strain = A/Puerto Rico/8/1934. Panel B depicts the alignment of 18 reads from one individual to a HCV subtype 3 sequence. Closest match, HCV clone FG1-NS3-4a from Pakistan (https://www.ncbi.nlm.nih.gov/nucleotide/KC825339). The number of reads represents and abundance is 912 HCV particles per 100,000 human cells. The viral reads are restricted to ~2Kb of the ~9Kb of HCV.
(TIF)

S6 Fig Association of viral presence with demographic characteristics. Panel A-C depict the individual association of viral presence with sex, age and genetic ancestry. Panel D plots the results of the analysis of deviance (variance) for the presence of any human virus in response to the individuals’ gender, ethnicity, age. AFR, African; AMR, Admixed American; EAS, East Asian; EUR, European; CSA, Central South Asian; MDE, Middle East.
(TIF)

S1 Table Complete listing of viruses putatively identified or contaminating blood DNA of 8,240 individuals. (PDF)

S2 Table Statistical significant differences for demographic characteristics and viral prevalence or viral load. (PDF)
