# Supplementary figures and images for: The blood DNA virome in 8,000 humans
Source: PLoS Pathog. 2017 Mar 22;13(3):e1006292. doi: 10.1371/journal.ppat.1006292 (PMC5378407; doi:10.1371/journal.ppat.1006292)

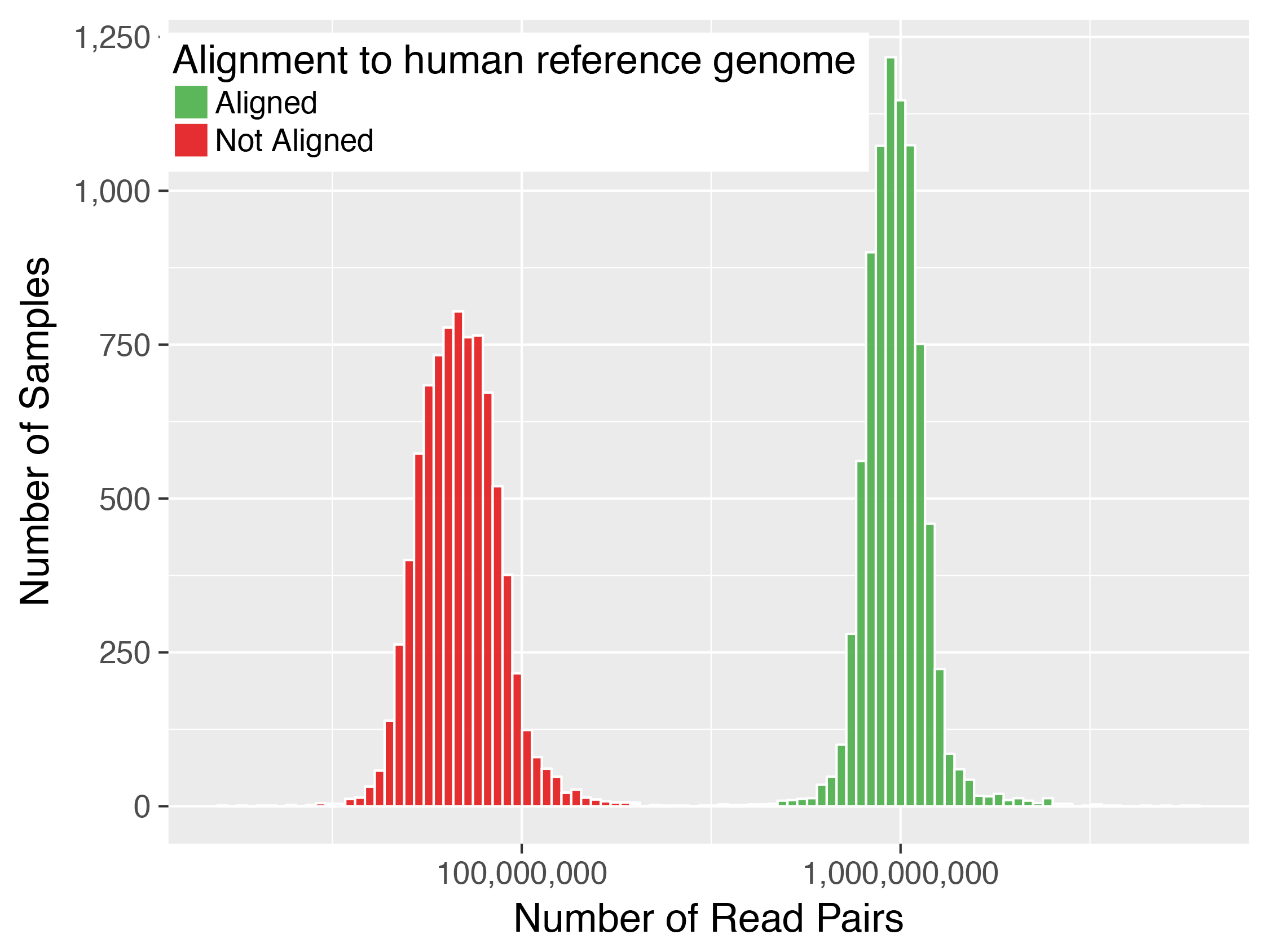

Supplement: S1 Fig — Unmapped reads in deep sequencing of the human genome using Illumina HiseqX10 technology. The average percentage of unmapped reads per sample is around 5.23%, and median is 4.91%. (TIF) [file ppat.1006292.s001.tif]

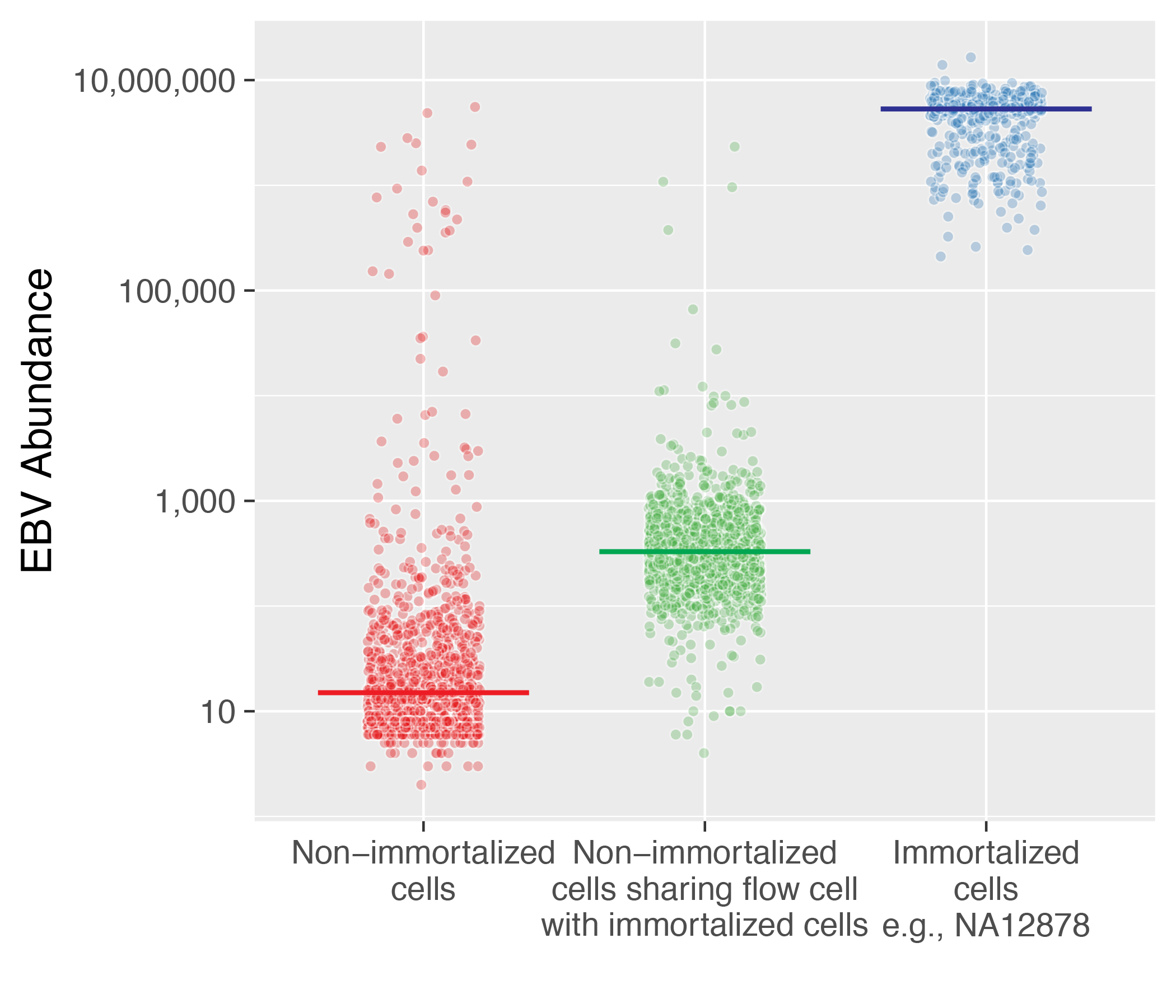

Supplement: S2 Fig — The distribution of the abundance of EBV is shown for the EBV B95-8 strain-immortalized the cell line of NA12878, for samples sequenced sharing the same flow cell with human genome NA12878 and for samples sequenced in the absence of human genome NA12878 in the sequencing flow cell. We used the conservative approach of eliminating all the positive samples from flow cells containing NA12879 because the high counts indicated that most samples were contaminated. Only a minority of samples had low counts, and we did not attempt alignment to the EBV B95-8 genome because of the few available reads. The bars represent the median. (TIF) [file ppat.1006292.s002.tif]

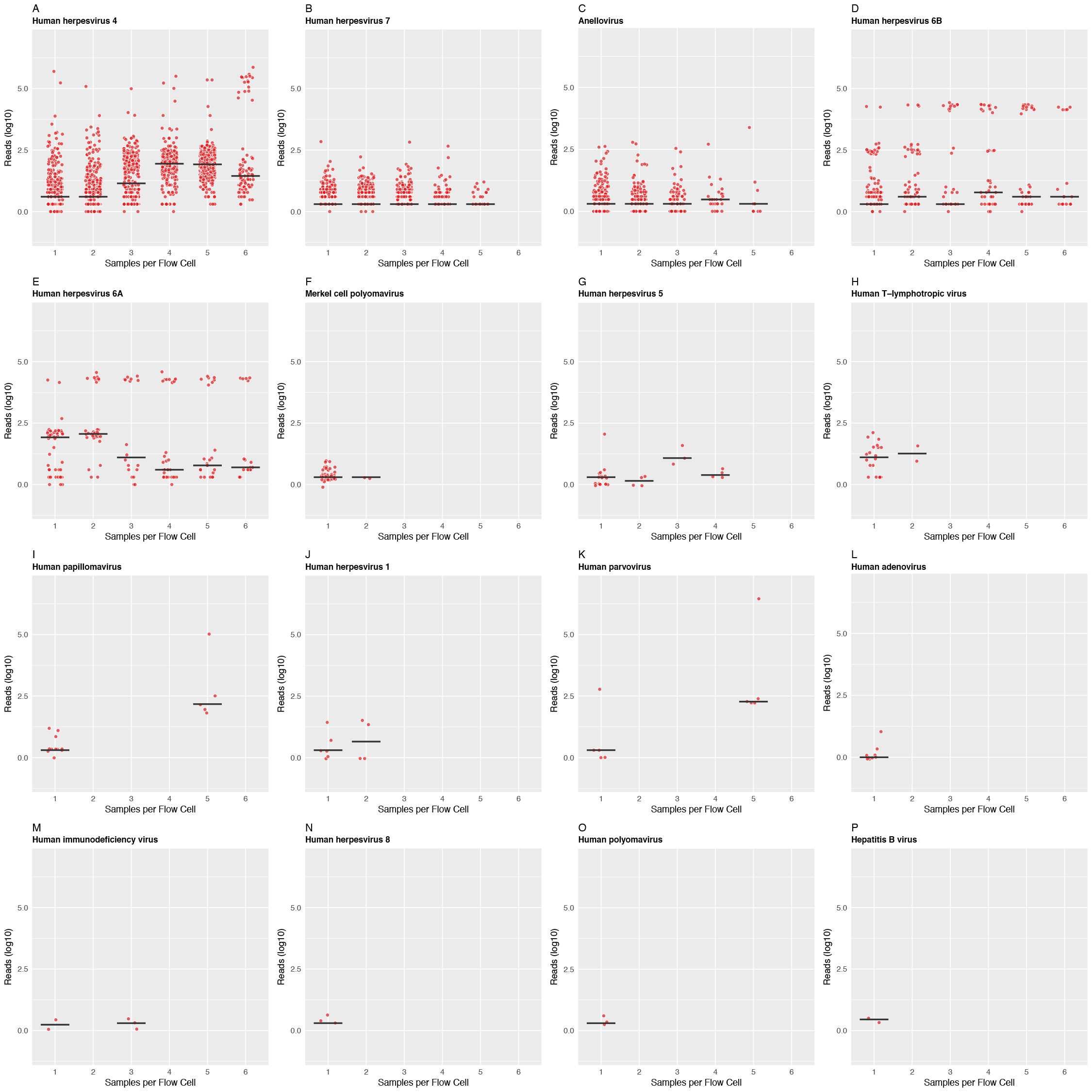

Supplement: S3 Fig — The number of viral reads per samples are shown on the y-axis in relation to the number of samples per flow cell that are positive for the corresponding virus. The presence of multiple positive samples in flow cells that contain one high viral-titer sample is suggestive of contamination by misidentification by sharing of barcodes in single-index sequencing libraries. The bars represent the median. (TIF) [file ppat.1006292.s003.tif]

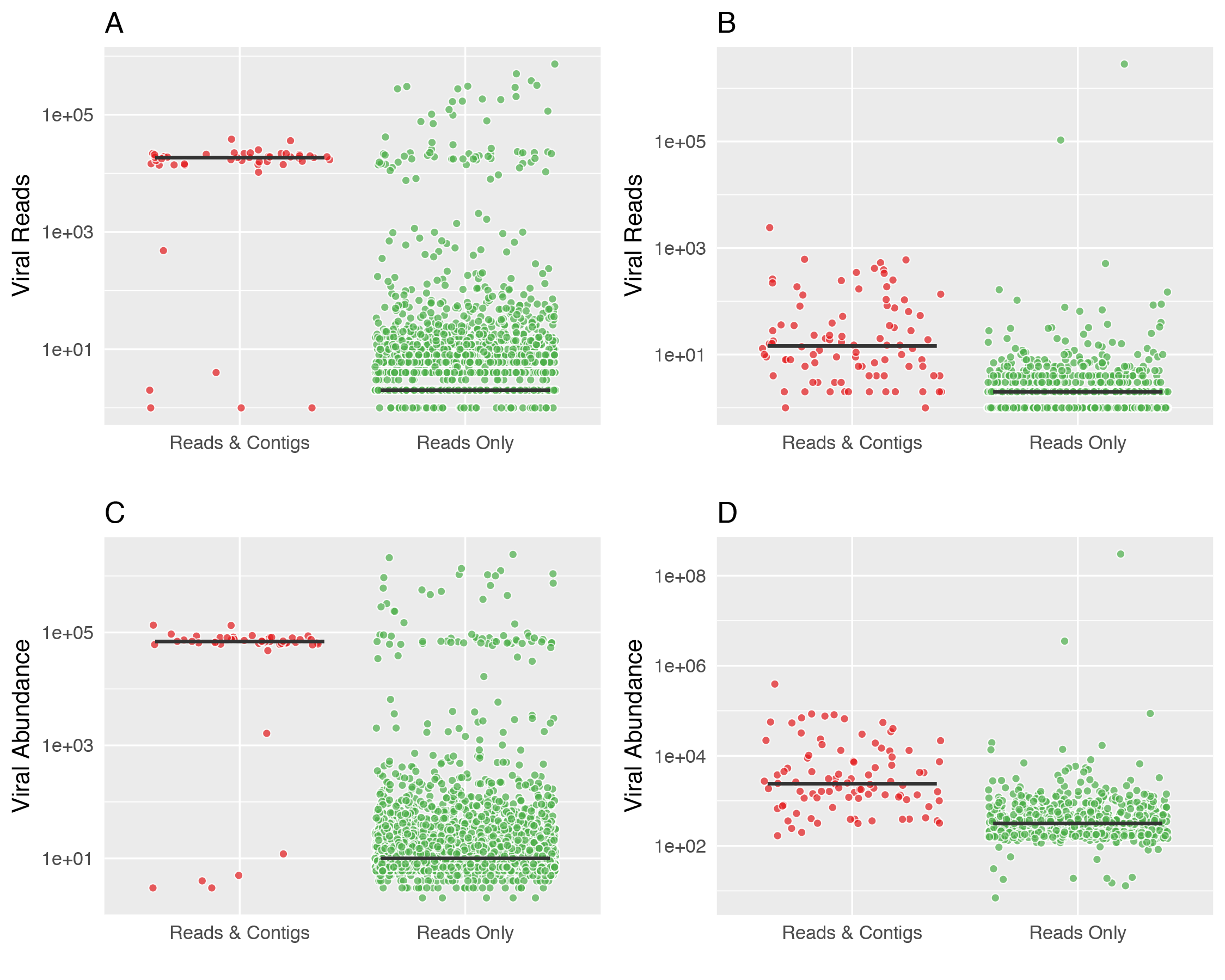

Supplement: S4 Fig — The sensitivity of identification of human viruses differs when using contigs from de novo assembly of reads, versus using individual reads. The upper panel is based on raw counts of the virus reads and the lower panels show the normalized viral abundances. The identification of viruses is improved by several orders when using read mapping. However, excessive number of reads (depth) may lead to failure of the assembly process Overall, viruses were detected by both read mapping or contigs in 137 samples, and only by read mapping in 3,342 samples. It came as a surprise that in 13 samples the identification of viral sequences (anellovirus, CMV, and HIV) was achieved using only contigs. After manual inspection, the CMV and HIV contigs represented plasmids sequences. Eleven samples with anelloviruses, represented by four clusters, were detected by contigs only because the individual reads had low identity (less than 70%) with the corresponding virus reference genome indicating the presence of divergent anelloviruses. Specifically, two contigs had the closest match as TTV-like mini LY1, one contig had the closest match as Torque teno mini virus 3, and one contig had the closest match as unclassified Anelloviridae isolate TPK01. The bars represent the median. (TIF) [file ppat.1006292.s004.tif]

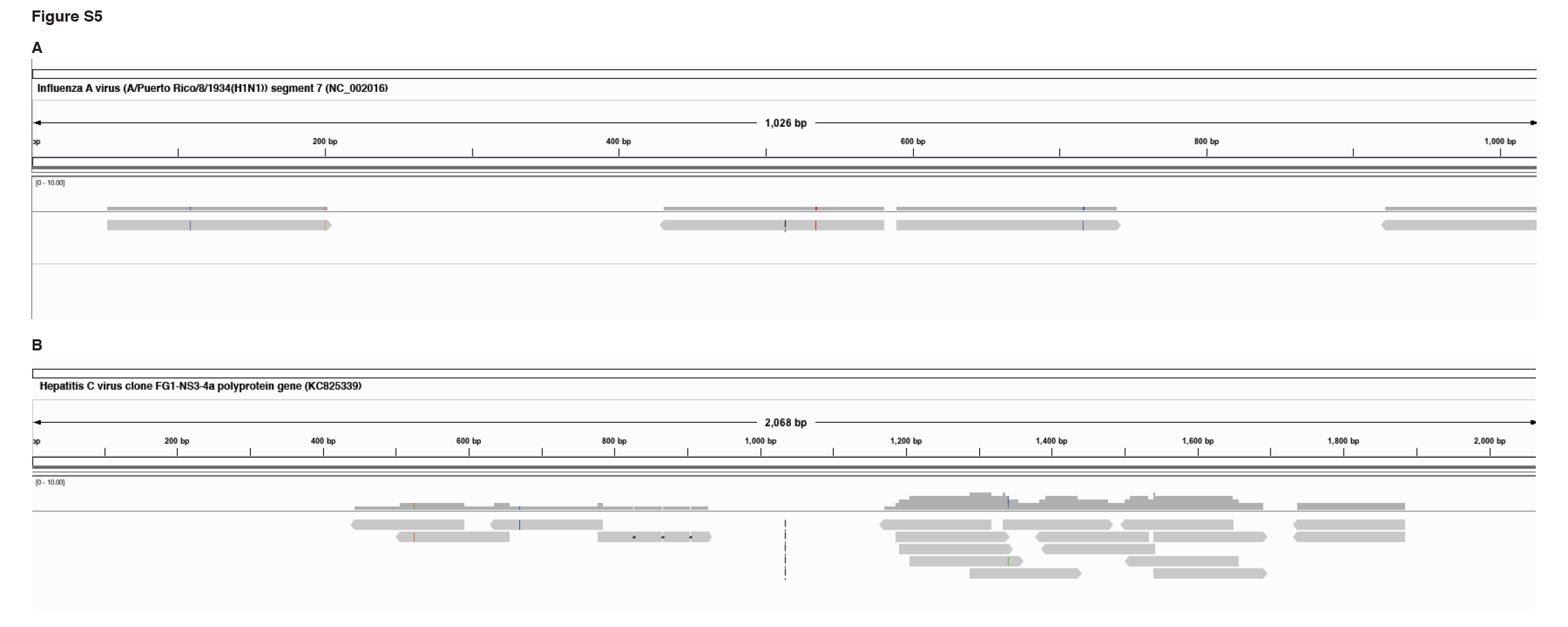

Supplement: S5 Fig — Panel A depicts the alignment of 4 reads from one individual to the influenza H1N1 reference sequence M1 and M2, segment seven. Closest match; serotype = H1N1, strain = A/Puerto Rico/8/1934. Panel B depicts the alignment of 18 reads from one individual to a HCV subtype 3 sequence. Closest match, HCV clone FG1-NS3-4a from Pakistan (https://www.ncbi.nlm.nih.gov/nucleotide/KC825339). The number of reads represents and abundance is 912 HCV particles per 100,000 human cells. The viral reads are restricted to ~2Kb of the ~9Kb of HCV. (TIF) [file ppat.1006292.s005.tif]

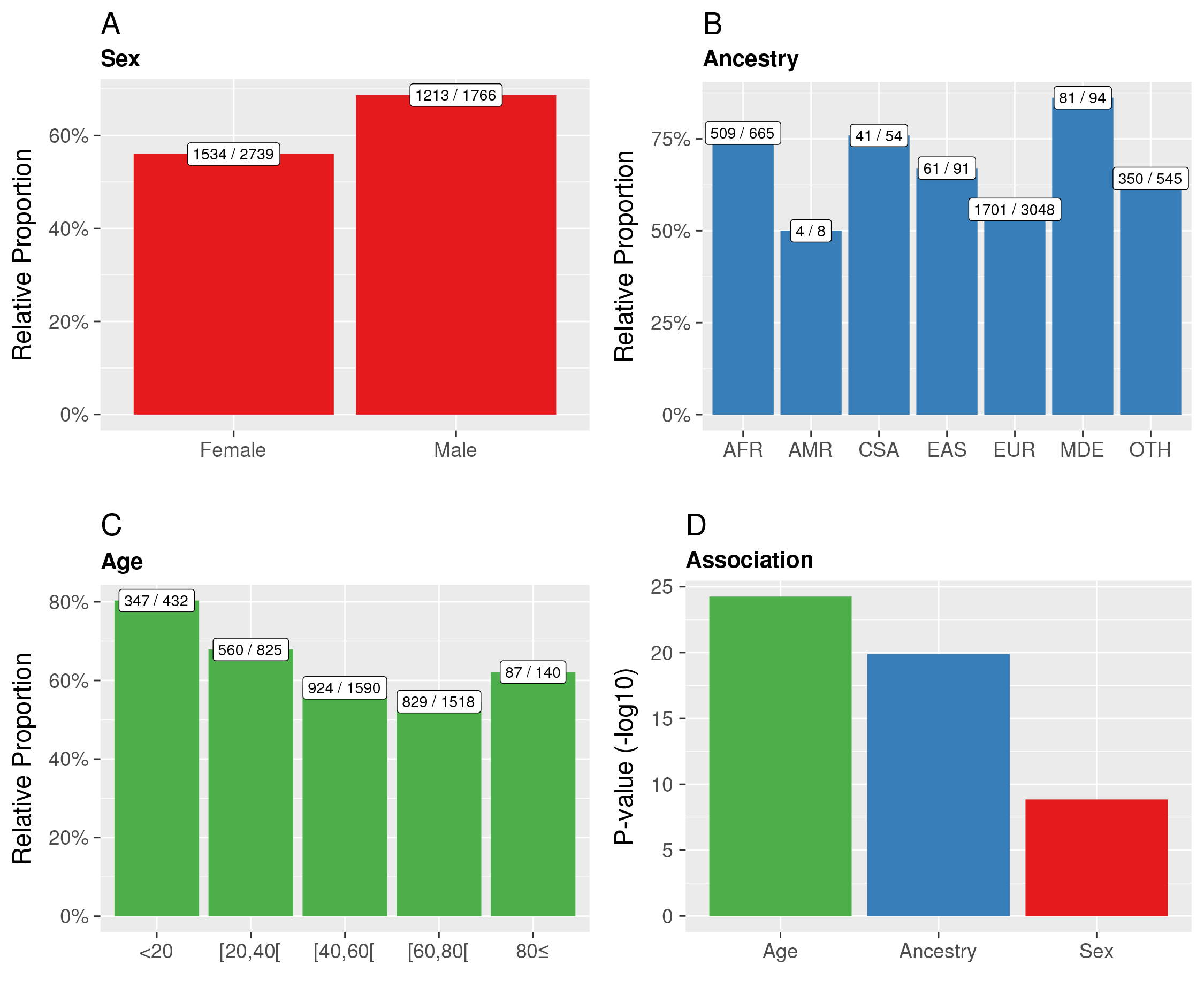

Supplement: S6 Fig — Panel A-C depict the individual association of viral presence with sex, age and genetic ancestry. Panel D plots the results of the analysis of deviance (variance) for the presence of any human virus in response to the individuals’ gender, ethnicity, age. AFR, African; AMR, Admixed American; EAS, East Asian; EUR, European; CSA, Central South Asian; MDE, Middle East. (TIF) [file ppat.1006292.s006.tif]
