# Supplementary material for: The blood DNA virome in 8,000 humans
Source: PLoS Pathog. 2017 Mar 22;13(3):e1006292. doi: 10.1371/journal.ppat.1006292 (PMC5378407; doi:10.1371/journal.ppat.1006292)
Supplement: S1 Table — (PDF) [file ppat.1006292.s007.pdf]

Table S1. Complete listing of viruses putatively identified or contaminating blood DNA of 8,240 individuals.

Virus	Number of individuals	Percentage of individuals	Median reads per individual	Maximum reads per individual	Median abundance	Maximum abundance
Human herpesvirus 7	1,679	20.38%	2	702	10	2,860
Human herpesvirus 4	1,192	14.47%	4	732,061	12	2,404,531
Enterobacteria phage	926	11.24%	2	502	28	6,763
Anellovirus	734	8.91%	2	2,416	359	392,179
Burkholderia phage	570	6.92%	3	199	49	2,746
Propionibacterium phage	566	6.87%	2	776	47	17,819
Human herpesvirus 6B	395	4.79%	2	26,738	9	97,274
Escherichia phage	384	4.66%	1	1,600	8	14,388
Ralstonia phage	360	4.37%	4	827	314	66,360
Stenotrophomonas phage	307	3.73%	2	294	69	10,479
Salmonella phage	287	3.48%	2	1,060	28	24,208
Pseudomonas phage	223	2.71%	2	6,216	29	89,728
Streptococcus phage	197	2.39%	5	61,622	76	1,103,637
Sinorhizobium phage	135	1.64%	1	69	14	733
Human herpesvirus 6A	121	1.47%	6	38,254	20	134,595
Rhizobium phage	118	1.43%	43	39,526	510	454,097
Staphylococcus phage	80	0.97%	2	424	29	5,417
Haemophilus phage	66	0.80%	2	7,886	42	147,447
Merkel cell polyomavirus	49	0.59%	2	8	236	935
Lactococcus phage	41	0.50%	2	1,857	39	38,658
Actinomyces phage	37	0.45%	1	643	74	17,953
Human herpesvirus 5	29	0.35%	2	106	5	338
Thermus phage	28	0.34%	2	41	9	181
Aggregatibacter phage	27	0.33%	1	76	17	982
Human T-lymphotropic virus	22	0.27%	13	131	820	13,143
Acinetobacter phage	21	0.25%	2	28	30	471
Lactobacillus phage	20	0.24%	2	338	32	5,519
Human papillomavirus	17	0.21%	2	106,590	163	3,521,083
Moloney murine leukemia virus	17	0.21%	1	2	74	177
Geobacillus phage	15	0.18%	2	32	31	524
Cronobacter phage	12	0.15%	1	3	4	12
Shigella phage	12	0.15%	1	91	7	822
Bacillus phage	11	0.13%	2	71	32	1,030
Citrobacter phage	10	0.12%	2	3	7	11

**Deep-sea thermophilic phage**	10	0.12%	2	60	26	815
**Human herpesvirus 1**	10	0.12%	2	34	9	123
**Human parvovirus**	10	0.12%	167	2,841,285	19,298	302,149,810
**Klebsiella phage**	10	0.12%	2	90	8	320
**Human adenovirus**	9	0.11%	1	11	19	235
**Mollivirus sibericum**	8	0.10%	2	4	2	4
**Achromobacter phage**	7	0.08%	1	3	9	30
**Agrobacterium phage**	6	0.07%	1	7	12	33
**Human immunodeficiency virus**	5	0.06%	2	3	142	275
**Archaeal virus**	4	0.05%	9	13	72	91
**Citrus endogenous pararetrovirus**	4	0.05%	1	1	94	118
**crAssphage**	4	0.05%	917	1,032	5,922	7,190
**Enterobacter phage**	4	0.05%	1	2	4	7
**Clostridium phage**	3	0.04%	4	7	35	88
**Delftia phage**	3	0.04%	1	2	8	19
**Human herpesvirus 8**	3	0.04%	2	4	8	17
**Human polyomavirus**	3	0.04%	2	4	297	588
**Mycobacterium phage**	3	0.04%	1	1	9	10
**Paramecium bursaria Chlorella virus**	3	0.04%	2	2	3	4
**Stx2-converting phage**	3	0.04%	1	58	11	565
**Thermus thermophilus phage**	3	0.04%	2	2	69	81
**Yellowstone lake phycodnavirus**	3	0.04%	2	2	7	8
**Yersinia phage**	3	0.04%	1	10	7	80
**Apis mellifera filamentous virus**	2	0.02%	2	2	2	2
**Ecotropic murine leukemia virus**	2	0.02%	37	74	3,248	6,443
**Enterococcus phage**	2	0.02%	7	14	83	159
**Hepatitis B virus**	2	0.02%	3	4	460	521
**Mannheimia phage**	2	0.02%	4	6	49	75
**Melbournevirus**	2	0.02%	3	4	5	7
**Ostreococcus virus**	2	0.02%	1	2	6	7
**Acanthamoeba polyphaga moumouvirus**	1	0.01%	2	2	1	1
**Aeromonas phage**	1	0.01%	1	1	17	17
**Bacteroides phage**	1	0.01%	2	2	29	29
**Bordetella phage**	1	0.01%	1	1	13	13
**Brochothrix phage**	1	0.01%	2	2	30	30

**Brucella phage**	1	0.01%	1	1	13	13
**Feline immunodeficiency virus**	1	0.01%	2	2	115	115
**Heliothis virescens ascovirus**	1	0.01%	2	2	8	8
**Hepatitis C virus**	1	0.01%	18	18	912	912
**Influenza A virus**	1	0.01%	4	4	3,128	3,128
**Invertebrate iridescent virus**	1	0.01%	2	2	4	4
**Microbacterium phage**	1	0.01%	1	1	12	12
**Murine osteosarcoma virus**	1	0.01%	20	20	3,781	3,781
**Papaya ringspot virus**	1	0.01%	4	4	228	228
**Parabacteroides phage**	1	0.01%	6	6	764	764
**Pectobacterium phage**	1	0.01%	1	1	6	6
**Pelagibacter phage**	1	0.01%	1	1	15	15
**Polytropic murine leukemia virus**	1	0.01%	102	102	8,958	8,958
**Porcine endogenous retrovirus**	1	0.01%	6	6	356	356
**RD114 retrovirus**	1	0.01%	2	2	155	155
**Serratia phage**	1	0.01%	2	2	35	35
**Sewage-associated gemycircularvirus**	1	0.01%	8	8	2,432	2,432
**Sindbis virus**	1	0.01%	2	2	80	80
**Spleen focus-forming virus**	1	0.01%	8	8	915	915
**Synechococcus phage**	1	0.01%	2	2	7	7
**Tobacco vein clearing virus**	1	0.01%	1	1	61	61
**Trichodysplasia spinulosa polyomavirus**	1	0.01%	2	2	219	219
**Tsukamurella phage**	1	0.01%	2	2	13	13
**Vibrio phage**	1	0.01%	2	2	28	28
**Xanthomonas phage**	1	0.01%	2	2	45	45
