# Supplementary material for: The blood DNA virome in 8,000 humans
Source: PLoS Pathog. 2017 Mar 22;13(3):e1006292. doi: 10.1371/journal.ppat.1006292 (PMC5378407; doi:10.1371/journal.ppat.1006292)
Supplement: S2 Table — (PDF) [file ppat.1006292.s008.pdf]

Table S2. Statistical significant differences for demographic characteristics and viral prevalence or viral load.

Virus	Sex		Ancestry		Age	
	Abundance	Prevalence	Abundance	Prevalence	Abundance	Prevalence
**EBV**			0.002			0.004
**CMV**						
**HHV6A**						
**HHV6B**	0.016		0.005	0.006		0.004
**HHV7**			1.04E-08	0.04	3.47E-12	0.004
**HTLV**				0.004		
**MCPyV**						
**Anellovirus**		0.004		0.004		0.004
